# Teeth and the Importance of Their Preservation

**Published:** 1865-07

**Authors:** J. S. Cobb


### TEETH AND THE IMPORTANCE OF THEIR PRESERVATION.

BY J. S. COBB, D. D. S.

From the little said upon this subject, in the many books, Periodicals, Pamphlets and Papers published, from time to time one would almost conclude that teeth are of minor importance, but upon investigation we find just the reverse. We find that they are of great importance. Hence, we propose to give a few plain simple words of advice not that we feel so able or competent to advise, but that we wish to call attention to the teeth; for we feel assured that there is no part of the human system more neglected than these valuable organs, in fact we know of nothing of as much importance, and more neglected and abused than the teeth. Take the people as a mass, and not more than one out of fifty give proper attention to their teeth. So taking all together, we find the above assertion true, (teeth are more neglected than any other part of the human system,) and why is this? Why so indifferent and unconcerned about our teeth. Why think so little of them? Why not look after and care for them as we would any other organ of the system? Let any other part of the system become diseased, and we soon become alarmed. We are ready to go to Doctoring. We are ready to advise and enquire of every one we meet what will do good. We are ready to take a thousand and one things. Why not, then, look after and care something for our teeth? Why not Doctor and try to restore them to health when they have become decayed; for we are sure there is nothing more calculated to derange and disease other organs of the system than a set of decayed teeth. Just think of the effect of a mouthful of decaying and vitiated matter, drawn by every breath upon the tender structure of the lungs, and circulated with the blood through the system. Producing diseases of various kinds. Just think of the pain and suffering man has to endure from allowing his mouth to become so unhealthy. Just think of the amount of disease that finds its way into the system through an unhealthy mouth. There is nothing plainer to one who has investigated the case, than that he who neglects his teeth and mouth, injures the whole system, for we can assure you that these things called teeth, are intimately connected with all the great functions of life, and that the human system is a delicately formed machine. Each part dependent upon the other, and sympathizing with the whole. So from nature's, chain whatever link we strike, great or small, brakes the chain alike; and whenever there is a morbid susceptibility of functional derangement, however little it may be, it will insidiously work out its deleterious effects in the end, and as the loss of one of these organs is a link from nature's chain, so is the tax increased on the remaining organs, not only of the mouth, but of the whole system. And continue, if you please, to let this morbid unhealthy action increase in the mouth, and not only the remainder of your teeth will go, but other organs of more tender vital power will fall a prey to desease, and from one to anothar until the last link of nature's chain will be severed, so we find by reasoning and investigating the matter, that it is important that teeth should be cared for, and well cared for too, for we feel assured that the preservation of our teeth adds more to the health than one out of a thousand has any idea of, but health is the principal thing that we wish to call attention to; what contributes more to man's pleasure and luxury while eating than a good sound set of grinders. What contributes more to his expression, either of face or language, than a clean nice, beautiful set of teeth. And we are sure, that every one will join us in saying that there is nothing that adds more to the beauty of a lady than a good nice clean set of these pearly gems, in fact, what is so necessary as to have the casket of the face studded with those priceless gems. A few more words and we are done. Being convinced as we are, that teeth are connected with all the great functions of life, and that nature has rendered them durable in their substance, combinding strength with symmetry, and being the hardest substance of our whole bodies, and intended by nature to survive every other part. We will just here put one question, with the privilege of answering. Why is it that we find these valuable organs decaying before middle age, and even in childhood's days. It is certainly from neglect; for we feel assured that nine hundred and ninety-nine out of a thousand could save their teeth, if they would use the means in their power; at all events they could save a very large majority through life, if they would commence in time.

How important it is for parents to teach their children to clean their teeth. Make it a regular habit with them. Teach them that teeth are valuable, they are of great importance they must be saved. Rear them up in this way and they will not depart from it.

We frequently hear people say, I don't believe it is worth while to pay any attention to my teeth, for they will rot out any how; we immediately tell them that they are certainly mistaken, for we feel assured that if they were kept literally clean with the assistance of a good, honest, skillful Dentist they might be saved through life? Why then suffer our mouths to become the receptacles of corruption and decay; for as above stated, health depends vastly upon the condition of the teeth and mouth.

